# A Simple Chaotic Map-Based Image Encryption System Using Both Plaintext Related Permutation and Diffusion

**DOI:** 10.3390/e20070535

**Published:** 2018-07-18

**Authors:** Linqing Huang, Shuting Cai, Mingqing Xiao, Xiaoming Xiong

**Affiliations:** 1 School of Automation, Guangdong University of Technology, Guangzhou 510006, China; 2 Department of Mathematics, Southern Illinois University, Carbondale, IL 62901, USA

**Keywords:** image cryptosystem, plaintext related, cat map, plaintext sensitivity

## Abstract

Recently, to conquer most non-plain related chaos-based image cryptosystems’ security flaws that cannot resist the powerful chosen/knownn plain-text attacks or differential attacks efficiently for less plaintext sensitivity, many plain related chaos-based image cryptosystems have been developed. Most cryptosystems that have adopted the traditional permutation–diffusion structure still have some drawbacks and security flaws: (1) most plaintext related image encryption schemes using only plaintext related confusion operation or only plaintext related diffusion operation relate to plaintext inadequately that cannot achieve high plaintext sensitivity; (2) in some algorithms, the generation of security key that needs to be sent to the receiver is determined by the original image, so these algorithms may not applicable to real-time image encryption; (3) most plaintext related image encryption schemes have less efficiency because more than one round permutation–diffusion operation is required to achieve high security. To obtain high security and efficiency, a simple chaotic based color image encryption system by using both plaintext related permutation and diffusion is presented in this paper. In our cryptosystem, the values of the parameters of cat map used in permutation stage are related to plain image and the parameters of cat map are also influenced by the diffusion operation. Thus, both the permutation stage and diffusion stage are related to plain images, which can obtain high key sensitivity and plaintext sensitivity to resist chosen/known plaintext attacks or differential attacks efficiently. Furthermore, only one round of plaintext related permutation and diffusion operation is performed to process the original image to obtain cipher image. Thus, the proposed scheme has high efficiency. Complete simulations are given and the simulation results prove the excellent security and efficiency of the proposed scheme.

## 1. Introduction

Nowadays, the security of confidential image or video has become increasingly important when the sensitive information is transmitted over public channels or stored in a third party. However, for the intrinsic features of digital images, such as bulky data capacity, high time redundancy and space redundancy, chaotic maps are suitable for image encryption because of their high complexity, sensitivity to initial conditions, infinite key space and random-like behavior, etc. [1,2,3,4,5,6,7,8,9,10,11,12,13,14,15,16,17,18,19]. For example, Ye et al. developed a simple image scrambling encryption algorithm based on a pixel bit that can change the position and gray value of pixel simultaneously [1]. In [2], the two-stage bit-level permutation algorithm is used to shuffle plain image, which can obtain a diffusion effect in the permutation stage. However, Li et al. pointed out that any encryption schemes using only permutation operation can be efficiently broken with $O(\left\lceil \log_{L}MN\right\rceil )$ plaintexts and $O(\left\lceil \log_{L}MN\right\rceil \times MN^{2})$ computational time. Thus, diffusion operation is necessary for security image encryption [20]. As early as 1998, Fridrich proposed a new symmetric block encryption using architecture which includes pixel-level permutation–diffusion structure that has drawn much attention [3]. Since then, many permutation–diffusion structure based image cryptosystems have been developed [4,5,6,7,8,9,10,11,12,13,14,15,16,17,18,19]. Because bit level permutation can achieve a confusion effect, bit level permutation is often used in some cryptosystems [4,5,6,7,8]. For example, a cryptosystem using expand-and-shrink scheme to permute the bit matrix decomposed from original image was proposed in [4], which has high efficiency. For a color plain image, the correlations between different channels are very high. Zhou et al. developed an image cryptosystem by using a skew tent map [6], whose three channels are transformed into a binary image and encrypted at the same time. Later in 2017, Chai et al. used Brownian motion to confuse the 8 bit planes decomposed from the original image, and then all permutated bit planes are converted into the permutated image. After a two-directional diffusion stage is performed to the permutated image, an encrypted image is obtained [8]. However, for the algorithm based on bit level permutation, in the permutation stage, the amount of data that needs to be processed is eight times as large as in pixel-level permutation based algorithms. Thus, most image encryption algorithms still adopt pixel-level permutation [9,10,11,12,13,14,15,16,17,18,19]. For instance, Chen et al. proposed an efficiency image cryptosystem using a lookup table constructed by chaotic systems in both pixel-level permutation and diffusion operation [10]. In [12], Chebyshev map and rotation equation are used in the encryption system’s confusion stage and diffusion stage, respectively, and detailed security analysis has been provided. Later in 2017, a new chaotic map based on Beta function is proposed and used in the generation of chaotic sequences that are used in an encryption process and the encryption scheme including permutation, diffusion and substitution operation has high security [17]. Furthermore, to obtain a high efficiency image encryption scheme, a confusion stage and diffusion stage are performed simultaneously using a chaotic map and DNA technique [19].

However, the permutation stage and diffusion stage are independent of the original image in most chaotic-based image encryption schemes mentioned above. Such schemes have the security flaws that the cryptosystem is insensitive to original images and secret keys and cannot resist chosen/known plaintext attacks or differential attacks, etc. Table 1 shows some typical approaches that have been used to attack some cryptosystems based on permutation–diffusion structure [21,22,23,24,25,26,27].

In order to conquer the issue of low key sensitivity and plaintext sensitivity, researchers have proposed some plaintext related image encryption schemes in recent years [28,29,30,31,32,33,34,35,36,37,38]. For some algorithms in [28,29,30,31,32], the confusion process is related to plain image in some ways. For instance, Liu et al. developed a half-pixel-level interchange permutation strategy in the permutation stage and the permutation stage is plain-image dependent, which can obtain high plaintext sensitivity [30]. In [32], Chai et al. developed a new permutation operation using random access bit-permutation mechanism, in which, the generation of key streams used in the permutation stage is related to plain image. For some algorithms in [33,34,35,36,37], the diffusion process is related to plain image. For example, in [36], combined with the characteristics of the original image and the chaotic sequences generated by the chaotic map, the key streams used in the diffusion stage are generated and related to plain image, which can achieve high sensitivity to the plain image. In [37], Li et al. developed a selective chaotic maps and DNA coding based image encryption system in which only four bits of each image pixel are encrypted using plain related diffusion. However, for other image encryption algorithms in [28,38], the generation of security keys that need to be sent to the receiver is determined by the original image, so these algorithms can achieve high plaintext sensitivity and excellent security performance. Based the fact that the security keys are changed, however, when encrypting different plain images, the encryption system may not be applicable to real-time image encryption, especially to real-time video encryption. Furthermore, for some algorithms based on plaintext-related mentioned above, there are some security drawbacks, such as high encryption time, low security key space, or not enough security to resist powerful chosen-plaintext. For instance, image cryptosystems developed in [33,34,35] have been analysed and broken with chosen plaintext attack in [39,40,41], respectively.

According to the analysis above, we propose a simple chaotic based color image encryption system using both plaintext related permutation and diffusion. The main novelties and contributions of the scheme are as follows:

(1) The proposed encryption system can be used to encrypt color images or gray images of any size. Some algorithms [5,8,9,10,15,29,31,33,35] mentioned above are developed to encrypt gray images. If these algorithms are used to encrypt R, G and B channels of original color image and then transform three encrypted gray imges into encrypted color image, the encryption system has less plaintext sensitivity because three channels of original color image are encrypted separately and do not have interaction in the encryption process. Furthermore, some other algorithms [7,10,11] are suitable for encrypting the original square image.
(2) As mentioned above, most plaintext related image encryption schemes used only plaintext related confusion operation [28,29,30,31,32] or only plaintext related diffusion operation [33,34,35,36,37] related to plaintext inadequately. For security purposes, in our scheme, both permutation operation and diffusion operation are related to plain images, which can achieve high plaintext sensitivity to chosen/known plaintext attack efficiently.
(3) Different from most chaotic based image cryptosystems in [4,5,6,7,9,10,12,16,19,28,29,31,36], in which the permutation–diffusion operation is performed several times to obtain the desired security level, the plaintext related permutation and diffusion in our scheme is only performed a single time in the entire encryption process.
(4) Complete simulations are given and the simulation results prove an excellent performance in security and efficiency.

The rest of this paper is organized as follows: two chaotic maps used in our image cryptosystem will be reviewed briefly in Section 2; Section 3 details the new encryption scheme; Section 4 gives detailed simulation to evaluate the performance of the new system; Section 5 provides conclusions.

## 2. Related Work

In our new chaotic encryption scheme, two chaotic maps are used and briefly discussed: extended Arnold map and Chebyshev map.

### 2.1. Extended Arnold Map

Cat map is a well-known two-dimensional invertible chaotic map and its extended version used to permutate non-square images in the permutation stage in our new cryptosystem is defined as the following equation:

(1) $$\left[\begin{array}{c} x^{\prime }\\ y^{\prime } \end{array}\right]=\left[\begin{array}{c} 1\\ b \end{array}\right.\left.\begin{array}{c} a\\ ab+1 \end{array}\right]\left[\begin{array}{c} x\\ y \end{array}\right]\;\mathrm{mod}\;\left[\begin{array}{c} M\\ N \end{array}\right],$$

where (x,y) and $\left(x^{\prime },y^{\prime }\right)$ are the current accessing position and the target position respectively, *a* and *b* are the parameters, and *M* and *N* are the height and the width of the plain image, respectively. When the target position $\left(x^{\prime },y^{\prime }\right)$ is obtained, two pixels located in (x,y) and $\left(x^{\prime },y^{\prime }\right)$ change places. Because $x,x^{\prime }=1,2,3\cdots M$, $y,y^{\prime }=1,2,3\cdots N$, actually, we use the following equation to permutate the original image:

(2) $$\left[\begin{array}{c} x^{\prime }\\ y^{\prime } \end{array}\right]=\left[\begin{array}{c} 1\\ b+1 \end{array}\right.\;\left.\begin{array}{c} a\\ a(b+1)+1 \end{array}\right]\left[\begin{array}{c} x\\ y \end{array}\right]\;\mathrm{mod}\;\left[\begin{array}{c} M\\ N \end{array}\right]+\left[\begin{array}{c} 1\\ 1 \end{array}\right].$$

### 2.2. Chebyshev Map

**Figure 1:**
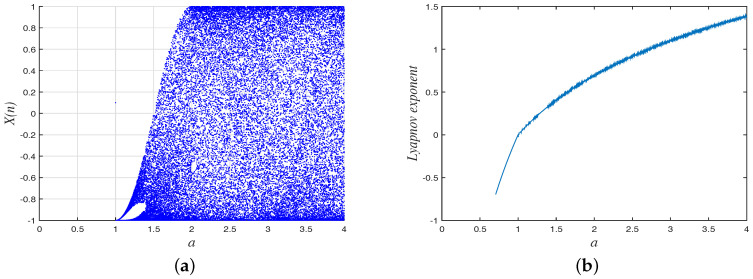
The Bifurcation diagram and Lyapunov Exponent diagram of the Chebyshev map. (**a**) Bifurcation diagram; (**b**) Lyapunov Exponent diagram.

For the advantages of a simple structure, ease of implementation and good chaotic performance, Chebyshev map is suitable for fast image encryption systems and have been used in many secure encryption systems [9,12,13,32]. The Chebyshev map is given by Equation (3):

(3) $$x_{n+1}=F\left(x_{n},a\right)=\cos\left(a\times arc\cos x_{n}\right),$$

where a∈N is the parameter and the output sequence is chaotic when a≥2. Giving the initial value $x_{0}$ of the sequence that can be used as secret key at a later stage, the chaotic sequence $x_{n}\in \left[-1,1\right]$ can be generated by the chaotic map. To measure the chaotic property of the Chebyshev Map, Bifurcation analysis and Lyapunov exponent analysis are given and the analysis results shown in Figure 1. As shown in Figure 1, the Chebyshev map has a chaotic behavior when parameter a≥2.

## 3. Algorithm of Image Encryption

**Figure 2:**
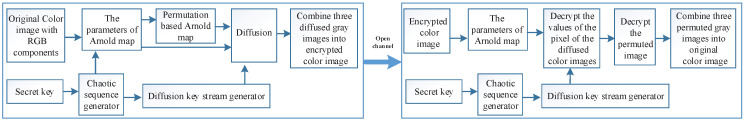
The proposed cryptosystem.

In this section, we detail the new encryption scheme adopting plaintext related traditional permutation–diffusion structure. The overall view of our new cryptosystem is shown in Figure 2.

### 3.1. Secret Key Formulation

There are five secret keys in our scheme including the initial value $key_{\_x0}$ of Chebyshev Map and four other positive integers $K_{1}$, $K_{2}$, $K_{3}$ and $N_{0}$ whose ranges are $key_{\_x0}\in \left(-1,1\right)$, $K_{1}\in \left[10^{5},10^{12}\right]$, $K_{2}\in \left[10^{5},10^{12}\right]$, $K_{3}\in \left[10^{5},10^{12}\right]$ and $N_{0}\in \left[1000,2500\right]$, respectively.

### 3.2. Encryption Process

The encryption process includes only one round permutation stage and diffusion stage.

#### 3.2.1. Permutation Stage

Step 1: The original color image *P* with size M×N×3 is divided into RGB (Red, Green, Blue) channels denoted as $P_{r}$, $P_{g}$ and $P_{b}$, respectively.

Step 2: The parameter and initial value of Chebyshev Map are set to a=4, and $key_{\_x0}=0.3$, respectively, and $N_{0}$ is defined as $N_{0}=1000$. Then, pre-iterate Equation (3) $\left(M\times N+N_{0}+9\right)$ times and discard the former $N_{0}$ elements to avoid the harmful effects to obtain random sequences $x_{n}$ with the size of (M×N+9), given by

(4) $$x_{n}=\left\{x_{1},x_{2},x_{3},\cdots ,x_{M\times N+9}\right\}.$$

The first nine elements of the sequence $x_{n}$ are processed using Equation (5) to obtain another sequence $x_{nq}$:

(5) $$x_{nq}\left(i\right)=\left(K_{1}⊗K_{2}⊗K_{3}\right)⊗floor\left(x_{n}\left(i\right)\times 10^{15}\right),$$

where i=1,2,3,…,9.

Step 3: Obtaining the sum of the pixels’ value in $P_{r}$, $P_{g}$ and $P_{b}$, respectively, one can get

(6) $$sum_{r}=\sum_{i=1}^{M}\sum_{j=1}^{N}P_{r}\left(i,j\right),$$

(7) $$sum_{g}=\sum_{i=1}^{M}\sum_{j=1}^{N}P_{g}\left(i,j\right),$$

(8) $$sum_{b}=\sum_{i=1}^{M}\sum_{j=1}^{N}P_{b}\left(i,j\right).$$

Step 4: Calculation of the parameters of cat map is related to plain image. In our encryption system, R, G and B channels are shuffled by cat map with different parameters and the parameters are calculated using the following equations:

(9) $$\left\{\begin{array}{c} b_{r}=\;\mathrm{mod}\;\left(x_{nq}\left(1\right)⊗sum_{r}+x_{nq}\left(2\right)⊗sum_{g}+x_{nq}\left(3\right)⊗sum_{b},256\right)\\ a_{r}=\;\mathrm{mod}\;\left(\left(b_{r}+1\right)\times \left(K_{1}⊗K_{2}⊗K_{3}\right),65,536\right)+1, \end{array}\right.$$

(10) $$\left\{\begin{array}{c} b_{g}=\;\mathrm{mod}\;\left(x_{nq}\left(4\right)⊗sum_{r}+x_{nq}\left(5\right)⊗sum_{g}+x_{nq}\left(6\right)⊗sum_{b},256\right)\\ a_{g}=\;\mathrm{mod}\;\left(\left(b_{g}+1\right)\times \left(K_{1}⊗K_{2}⊗K_{3}\right),65,536\right)+1, \end{array}\right.$$

(11) $$\left\{\begin{array}{c} b_{b}=\;\mathrm{mod}\;\left(x_{nq}\left(7\right)⊗sum_{r}+x_{nq}\left(8\right)⊗sum_{g}+x_{nq}\left(9\right)⊗sum_{b},256\right)\\ a_{b}=\;\mathrm{mod}\;\left(\left(b_{b}+1\right)\times \left(K_{1}⊗K_{2}⊗K_{3}\right),65,536\right)+1, \end{array}\right.$$

where $\left(b_{r},a_{r}\right)$, $\left(b_{g},a_{g}\right)$ and $\left(b_{b},a_{b}\right)$ are the parameters of cat map used to shuffle R channel, G channel and B channel, respectively. If all pixels’ value in original color image *P* are zero, the parameters are calculated using the following equation:

(12) $$\left\{\begin{array}{c} b_{r}=\;\mathrm{mod}\;\left(x_{nq}\left(1\right)⊗x_{nq}\left(2\right)⊗x_{nq}\left(3\right),256\right)\\ a_{r}=\;\mathrm{mod}\;\left(\left(b_{r}+1\right)\times \left(K_{1}⊗K_{2}⊗K_{3}\right),65,536\right)+1, \end{array}\right.$$

(13) $$\left\{\begin{array}{c} b_{g}=\;\mathrm{mod}\;\left(x_{nq}\left(4\right)⊗x_{nq}\left(5\right)⊗x_{nq}\left(6\right),256\right)\\ a_{g}=\;\mathrm{mod}\;\left(\left(b_{g}+1\right)\times \left(K_{1}⊗K_{2}⊗K_{3}\right),65,536\right)+1, \end{array}\right.$$

(14) $$\left\{\begin{array}{c} b_{b}=\;\mathrm{mod}\;\left(x_{nq}\left(7\right)⊗x_{nq}\left(8\right)⊗x_{nq}\left(9\right),256\right)\\ a_{b}=\;\mathrm{mod}\;\left(\left(b_{b}+1\right)\times \left(K_{1}⊗K_{2}⊗K_{3}\right),65,536\right)+1. \end{array}\right.$$

**Figure 3:**
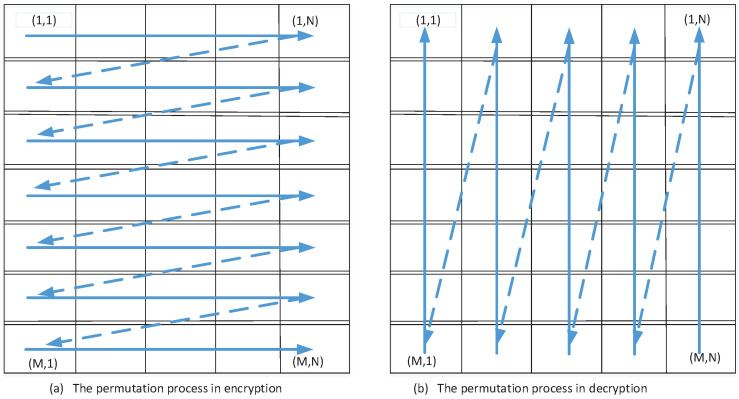
The scanning process in the permutation stage in encryption and decryption.

Step 5: Plaintext related permutation operation based on a cat map. All channels of original color image *P* are shuffled used Equation (2). The scanning sequence is left to right and top to bottom, which is illustrated in Figure 3a. When all pixels in R, G or B channels are permutated, the permutated image $P_{P}$ is obtained.

#### 3.2.2. Diffusion Stage

Step 1: Three permutated R, G and B channels gray images obtained in the permutation stage are transformed into three 1D pixel arrays ($P_{R\_p}$, $P_{G\_p}$, $P_{B\_p}$), respectively. One can get

(15) $$\left\{\begin{array}{c} P_{R\_p}=\left\{{p_{R\_p}}_{1},{p_{R\_p}}_{2},{p_{R\_p}}_{3},\cdots ,{p_{R\_p}}_{\left(M\times N\right)}\right\},\\ P_{G\_p}=\left\{{p_{G\_p}}_{1},{p_{G\_p}}_{2},{p_{G\_p}}_{3},\cdots ,{p_{G\_p}}_{\left(M\times N\right)}\right\},\\ P_{B\_p}=\left\{{p_{B\_p}}_{1},{p_{B\_p}}_{2},{p_{B\_p}}_{3},\cdots ,{p_{B\_p}}_{\left(M\times N\right)}\right\}. \end{array}\right.$$

Step 2: Using the random sequences $x_{n}$, the diffusion matrix $D=\{d_{1},d_{2},d_{3},\cdots ,d_{\left(M\times N\right)}\}$ is obtained, given by

(16) $$D\left(i\right)=\;\mathrm{mod}\;(floor\left(x_{n}\left(i+10\right)\times \left(K_{1}⊗K_{2}⊗K_{3}\right)\right),256),$$

where i=1,2,3,⋯,M×N.

Step 3: Calculation of $C_{R}\left(1\right)$, $C_{G}\left(1\right)$, $C_{B}\left(1\right)$ value. Here, three 1D pixel arrays $P_{R\_p}$, $P_{G\_p}$ and $P_{B\_p}$ are diffused to obtain the corresponding diffused 1D pixel arrays $C_{R}$, $C_{G}$ and $C_{B}$, respectively. The values of the first encrypted pixel $C_{R}\left(1\right)$, $C_{G}\left(1\right)$ and $C_{B}\left(1\right)$ are determined by the parameter of *b* and secret keys $K_{1}$, $K_{2}$, $K_{3}$ according the following equations:

(17) $$\left\{\begin{array}{c} C_{R}\left(1\right)=\;\mathrm{mod}\;\left(b_{r}+K_{1},256\right),\\ C_{G}\left(1\right)=\;\mathrm{mod}\;\left(b_{g}+K_{2},256\right),\\ C_{B}\left(1\right)=\;\mathrm{mod}\;\left(b_{b}+K_{3},256\right). \end{array}\right.$$

Step 3: Using the diffusion matrix *D* and 1D pixel arrays $P_{R\_p},P_{G\_p},P_{B\_p}$, all the other encrypted pixels of 1D pixel arrays $C_{R}$, $C_{G}$ and $C_{B}$ except $C_{R}\left(1\right)$, $C_{G}\left(1\right)$ and $C_{B}\left(1\right)$ are obtained, given by

(18) $$\left\{\begin{array}{c} C_{R}\left(i\right)=\;\mathrm{mod}\;\left(P_{R\_P}\left(i\right)⊗D\left(i\right)+num,256\right)⊗C_{R}\left(i-1\right),\\ C_{G}\left(i\right)=\;\mathrm{mod}\;\left(P_{G\_P}\left(i\right)⊗D\left(i\right)+num,256\right)⊗C_{G}\left(i-1\right),\\ C_{B}\left(i\right)=\;\mathrm{mod}\;\left(P_{B\_P}\left(i\right)⊗D\left(i\right)+num,256\right)⊗C_{B}\left(i-1\right), \end{array}\right.$$

where $num=\left(a_{r}\times b_{r}+a_{g}\times b_{g}+a_{b}\times b_{b}\right)⊗\left(K_{1}+K_{2}+K_{3}\right)$, i=2,3,4,⋯,M×N, and symbol “⊗” is bitwise exclusive or an operator.

Step 4: Convert the three 1D pixel arrays $C_{R}$, $C_{G}$ and $C_{B}$ into R, G and B channels gray images with the size of M×N, respectively. Then, three gray images are treated as RGB components of the last encrypted color image *C* with size M×N×3.

It should be noted that, if the encryption system is used to encrypt a gray image, then the encryption process is similar to the R, G or B channels encryption except the calculation of the first encrypted pixel C(1). Here, we use $C\left(1\right)=\;\mathrm{mod}\;(b_{r}+\left(K_{1}⊗K_{2}⊗K_{3}\right),256)$ to calculate the first encrypted pixel C(1).

### 3.3. Decryption Process

As illustrated in Figure 2, when the receiver obtains the encrypted image and secret keys $key_{\_x0}$, $K_{1}$, $K_{2}$, $K_{3}$ and $N_{0}$, the decryption process contains the following steps:

Step 1: Obtain the diffusion matrix $D=\{d_{1},d_{2},d_{3},\cdots ,d_{\left(M\times N\right)}\}$ using the same methods in the encryption process.

Step 2: The first encrypted pixels $C_{R}\left(1\right)$, $C_{G}\left(1\right)$, $C_{B}\left(1\right)$ are read from encrypted color image *C* and used to calculate the parameters of cat map as follows:

(19) $$\left\{\begin{array}{c} b_{r}=\;\mathrm{mod}\;\left(C_{R}\left(1\right)+256-K_{1},256\right),\\ b_{g}=\;\mathrm{mod}\;\left(C_{G}\left(1\right)+256-K_{2},256\right),\\ b_{b}=\;\mathrm{mod}\;\left(C_{B}\left(1\right)+256-K_{3},256\right), \end{array}\right.$$

(20) $$\left\{\begin{array}{c} a_{r}=\;\mathrm{mod}\;\left(\left(b_{r}+1\right)\times \left(K_{1}⊗K_{2}⊗K_{3}\right),65,536\right)+1,\\ a_{g}=\;\mathrm{mod}\;\left(\left(b_{g}+1\right)\times \left(K_{1}⊗K_{2}⊗K_{3}\right),65,536\right)+1,\\ a_{b}=\;\mathrm{mod}\;\left(\left(b_{b}+1\right)\times \left(K_{1}⊗K_{2}⊗K_{3}\right),65,536\right)+1, \end{array}\right.$$

Step 3: Reconstruct R, G and B channels of the permutated image $P_{P}$ using the diffusion equation as

(21) $$\left\{\begin{array}{c} P_{R\_p}\left(i\right)=\;\mathrm{mod}\;\left(C_{R}\left(i\right)⊗C_{R}\left(i-1\right)+256-num,256\right)⊗D\left(i\right),\\ P_{G\_p}\left(i\right)=\;\mathrm{mod}\;\left(C_{G}\left(i\right)⊗C_{G}\left(i-1\right)+256-num,256\right)⊗D\left(i\right),\\ P_{B\_p}\left(i\right)=\;\mathrm{mod}\;\left(C_{B}\left(i\right)⊗C_{B}\left(i-1\right)+256-num,256\right)⊗D\left(i\right), \end{array}\right.$$

Step 4: Reconstruct R, G and B channels of original color image *P* according to Equation (2). However, it should be noted that the scanning sequence of the accessing position is right to left and bottom to top, which is illustrated in Figure 3b.

## 4. Experimental Results and Security Analysis

**Figure 4:**
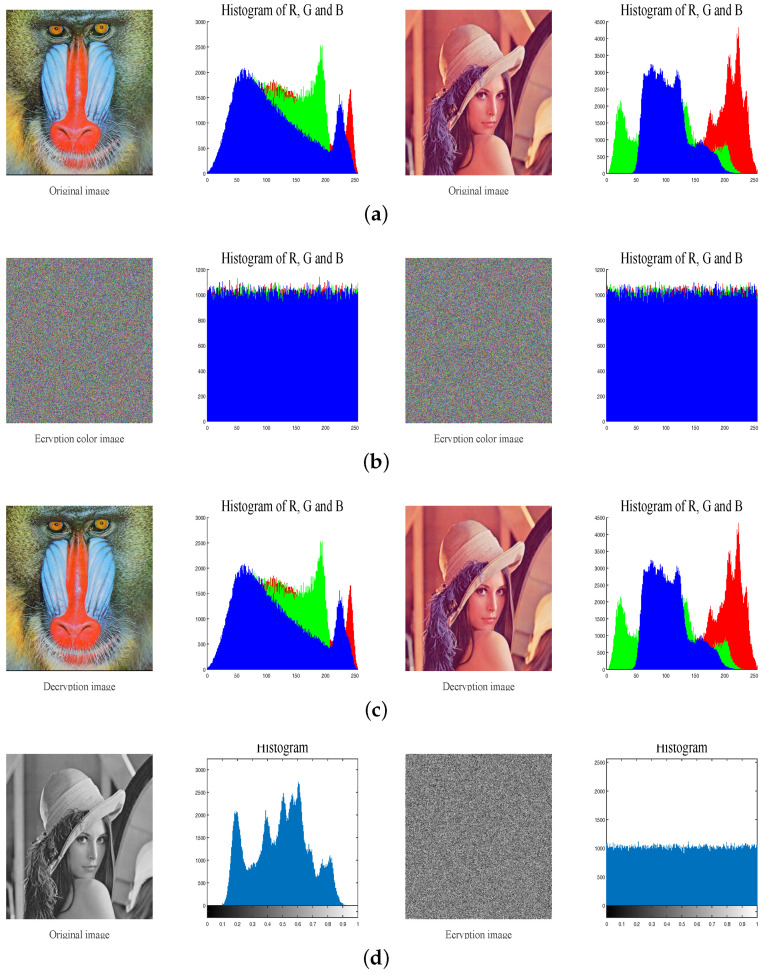
Encryption and decryption result of two images. (**a**) The plain-image and corresponding histograms; (**b**) The cipher images and corresponding histograms; (**c**) The decrypted images and corresponding histograms; (**d**) The gray original image and encrypted image and they corresponding histograms.

To test the performance of the proposed image cryptosystem, we choose two standard color plain images as the testing images. The initial value of Chebyshev map is chosen as $key_{\_x0}=0.3$ while the other four keys $K_{1}$, $K_{2}$, $K_{3}$ and $N_{0}$ are chosen as 65,536, 65,535, 65,534 and 1000, respectively. The comparison results of plain-images, encryption-decryption images and their corresponding histograms are shown in Figure 4.

### 4.1. Security Key Space

A security image cryptosystem should have large enough key space to resist brute force attack effectively. The ranges of five secret keys in our encryption system are that $key_{\_x0}\in \left(-1,1\right)$, $K_{1}\in \left[10^{5},10^{12}\right]$, $K_{2}\in \left[10^{5},10^{12}\right]$, $K_{3}\in \left[10^{5},10^{12}\right]$ and $N_{0}\in \left[1000,2500\right]$. If the precision of $key_{\_x0}$ is $10^{16}$, the key space will reach $key_{total}=10^{16}\times \left(10^{12}-10^{5}\right)\times \left(10^{12}-10^{5}\right)\times \left(10^{12}-10^{5}\right)\times 1500\approx 2^{183}$, which is more than $2^{128}$. Apparently, the cryptosystem is secure when facing brute-force attacks.

### 4.2. Histogram Analysis

Image histogram, which can provide attackers with the statistical information of the image, reflects the distribution of pixels’ value. For a security image cryptosystem, the cipher image’s histogram should be flat to resist statistic attacks. As can seen in Figure 4, completely different from the plain image’s histogram, the histogram of encrypted image is uniformly distributed.

For quantity analysis of the uniformity of image histogram, we use a variance of an image histogram that is presented as follows to evaluate the uniformity of image histogram:

(22) $$\mathrm{var}\left(H\right)=\frac{\sum_{i=0}^{255}{\left(h_{i}-\bar{H}\right)}^{2}}{256},$$

where image histogram $H=\left\{h_{1},h_{2},\cdots ,h_{256}\right\}$ is a vector, and $h_{i}$ is the value of histograms. The smaller the value of variance, the flatter the image histogram. Table 2 lists the variances of histograms of some ciphered test images. As shown in Table 2, the value of variance is very small, which means that the histogram of cipher image has very small average fluctuation.

### 4.3. Correlation Analysis

Adjacent pixels in original images often have high correlation, which can be used in statistical analysis attacks. Thus, after an original image is encrypted, its correlation coefficients of adjacent pixels should be greatly reduced. As a test, the correlation coefficients of all adjacent pixels in the four directions, including the vertical, horizontal, diagonal and anti-diagonal directions are calculated using Equation (23):

(23) $$r_{xy}=\frac{\mathrm{cov}(x,y)}{\sqrt{D(x)}\times \sqrt{D(y)}},$$

where $\mathrm{cov}\left(x,y\right)=\frac{1}{N}\sum_{i=0}^{N}(x_{i}-E\left(x\right))\left(y_{i}-E\left(y\right)\right)$, $D\left(x\right)=\frac{1}{N}\sum_{i=0}^{N}(x_{i}-E\left(x\right){)}^{2}$, E(x)$=\frac{1}{N}\sum_{i=0}^{N}x_{i}$. x,y are two adjacent pixel values, and *N* is the number of image pixel.

As Table 3 shows, all correlation coefficients of cipher-images including four directions, namely, vertical (V), horizontal (H), diagonal (D) and anti-diagonal (A), are almost equal to 0. Thus, the proposed scheme has excellent performance in terms of resisting statistical attack. Furthermore, Table 4 gives detailed results compared with similar schemes.

**Figure 5:**
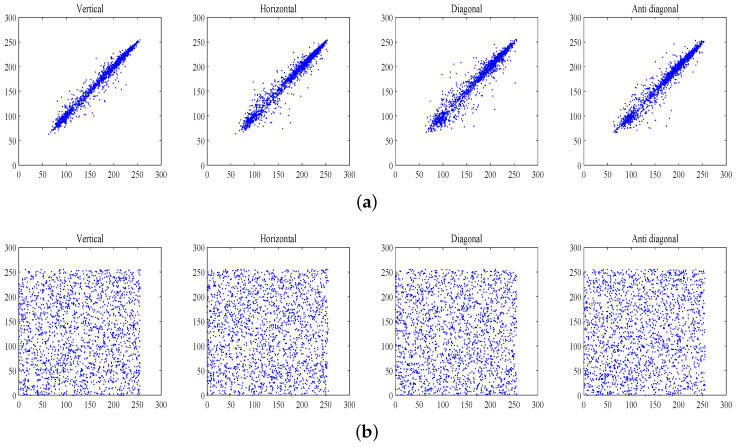
Correlation of R channel in standard “Lena” image. (**a**) Correlation of R channel in plain image; (**b**) Correlation of R channel in encrypted image.

Furthermore, 2000 pairs adjacent pixels in different directions are selected randomly from the R channel of a standard image of Lena and its corresponding encrypted image and Figure 5 shows the correlation diagram. As Figure 5 shows, adjacent pixels in cipher-images have less correlation.

### 4.4. Sensitivity Analysis

Differential attack is a powerful typical approach to break a cryptosystem. To resist such attack effectively, a security encryption system should have high key sensitivity and plaintext sensitivity. Two indexes, namely number of pixels change rate (NPCR) and unified average changing intensity (UACI), are defined as follows and used to evaluate the sensitivity:

(24) $$\left\{\begin{array}{c} NPCR=\sum_{i=0}^{H}\sum_{j=0}^{W}D(i,j)\times 100\%,\\ UACI=\frac{1}{W\times H}\sum_{i=0}^{H}\sum_{j=0}^{W}\frac{\left|c_{1}\left(i,j\right)-c_{2}\left(i,j\right)\right|}{255}\times 100\%, \end{array}\right.$$

where $c_{1},c_{2}$ are encrypted images, and $D\left(i,j\right)=\left\{\begin{array}{c} 0,\;if\;c_{1}\left(i,j\right)=c_{2}\left(i,j\right),\\ 1,\;if\;c_{1}\left(i,j\right)\neq c_{2}\left(i,j\right). \end{array}\right.$

There are five secret keys $key_{\_x0}$, $K_{1}$, $K_{2}$, $K_{3}$ and $N_{0}$ in our algorithm and we take secret key $key_{\_x0}$ as an example to illustrate the key sensitivity simulation. Firstly, 200 key groups $k_{ey}\left(i\right)=\left(key_{\_x0}\left(i\right),K_{1}\left(i\right),K_{2}\left(i\right),K_{3}\left(i\right),N_{0}\left(i\right)\right)\left(i=1,2,3,\cdots ,200\right)$ are selected from the security key space randomly and used to encrypt the standard plain-images to obtain 200 cipher-images denoted as $C_{1}\left(i\right)\left(i=1,2,3,\cdots ,200\right)$. Secondly, secret key $key_{\_x0}$ in each key group make a tiny change of $10^{-15}$ and the remaining four keys $K_{1}\left(i\right),K_{2}\left(i\right),K_{3}\left(i\right)andN_{0}$ keep unchanged to obtain 200 new key groups $k_{ey}\left(i\right)=\left(key_{\_x0}\left(i\right)+10^{-15},K_{1}\left(i\right),K_{2}\left(i\right),K_{3}\left(i\right),N_{0}\left(i\right)\right)\left(i=1,2,3,\cdots ,200\right)$. Then, the new 200 key groups are used to encrypt the same standard plain-images to obtain another 200 cipher-images denoted as $C_{2}\left(i\right)\left(i=1,2,3,\cdots ,200\right)$. Finally, using $C_{1}\left(i\right)\left(i=1,2,3,\cdots ,200\right)$ and $C_{2}\left(i\right)\left(i=1,2,3,\cdots ,200\right)$, 200 pairs of NPCR and UACI are calculated according to Equation (24). Table 5 shows average values of NPCR and UACI. The key sensitivity of $K_{1}$, $K_{2}$, $K_{3}$ and $N_{0}$ is evaluated in the same way and it should be noted that the variation of $K_{1}$, $K_{2}$, $K_{3}$ and $N_{0}$ is equal to 1. As Table 5 shows, the mean values of NPCR and UACI are almost equal to the theoretical value, which represents that our scheme has high key sensitivity.

**Figure 6:**
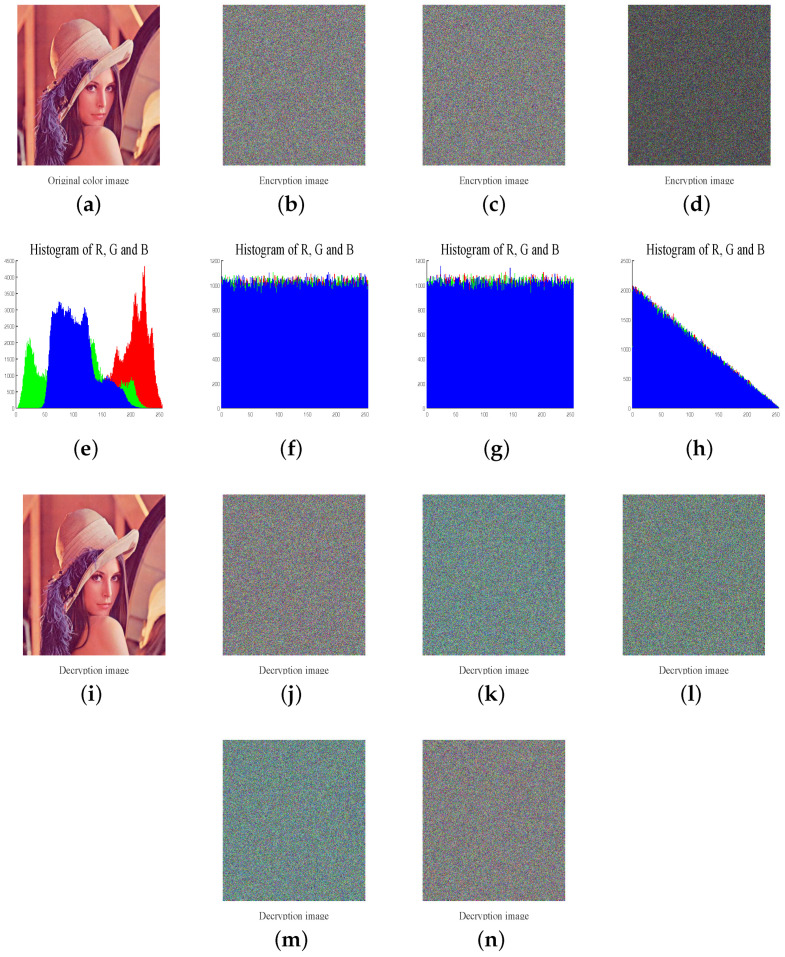
Key sensitivity tests: (**a**,**e**): plain-image and its histogram; (**b**,**f**): E1 and its histogram; (**c**,**g**): E2 and its histograms; (**d**,**h**): |E1−E2| and its histogram; (**i**): decrypted image of E1 using key set $k_{ey}\left(1\right)$ ; (**j**–**n**): decrypted image of E1 using security key set $k_{ey}\left(2\right)$, $k_{ey}\left(3\right)$, $k_{ey}\left(4\right)$, $k_{ey}\left(5\right)$ or $k_{ey}\left(6\right)$.

Furthermore, we use a standard image of Lena as a testing image and take secret key $key_{\_x0}$ as an example to show the key sensitivity test result visually. Firstly, one key group $k_{ey}\left(1\right)=\left(0.3,65,536,65,535,65,534,1000\right)$ is selected from the key space and used to encrypt the standard image in Figure 6a to obtain a cipher image denoted as E1 shown in Figure 6b. Then, the value of $key_{\_x0}$ is changed by $10^{-16}$ while keeping others unchanged to obtain another key group denoted as $k_{ey}\left(2\right)=\left(0.3000000000000001,65,536,65,535,65,534,1000\right)$. The key group $k_{ey}\left(2\right)$ is used to encrypt the same standard image to obtain another encrypted image denoted as E2 shown in Figure 6c. The image of pixel-to-pixel difference |E1−E2| and its histogram are shown in Figure 6d,h, from which we can see that a slight change $10^{-16}$ in secret key $key_{\_x0}$ will result in a significant change in the encrypted image. Finally, we obtain four other key sets $k_{ey}\left(3\right)=\left(0.3,65,537,65,535,65,534,1000\right)$, $k_{ey}\left(4\right)=\left(0.3,65,536,65,536,65,534,1000\right)$, $k_{ey}\left(5\right)=\left(0.3,65,536,65,535,65,535,1000\right)$ and $k_{ey}\left(6\right)=\left(0.3,65,536,65,535,65,534,1001\right)$ in the same way. After that, the decrypted image of cipher images E1 using six key sets $k_{ey}\left(1\right)$, $k_{ey}\left(2\right)$, $k_{ey}\left(3\right)$, $k_{ey}\left(4\right)$, $k_{ey}\left(5\right)$ and $k_{ey}\left(6\right)$ are shown in Figure 6i to Figure 6m. As one can see, only correct key set $k_{ey}\left(1\right)$ can reconstruct the original image absolutely.

An encryption system that can resist known and chosen plaintext attacks must be sensitive to tiny differences in the original image. Firstly, we used one key set denoted as $k_{ey}=\left(key_{\_x0},K_{1},K_{2},K_{3},N_{0}\right),$ which is selected from the security key space randomly to encrypt the standard plain-image to obtain an encrypted image denoted as $C_{1}^{\prime }$. Then, one pixel $P_{ixel\_1}\left(x,y,z\right)$ is selected from the standard plain-images randomly and modified its value slightly according to Equation (25):

(25) $$P_{ixel}\left(x,y,z\right)=\;\mathrm{mod}\;\left(P_{ixel}\left(x,y,z\right)+1,256\right).$$

After that, the standard plain-image containing the modified pixel $P_{ixel\_1}\left(x,y,z\right)$ is encrypted using key group $k_{ey}$ to obtain another cipher image $C_{2}^{\prime }$. Finally, using $C_{1}^{\prime }$ and $C_{2}^{\prime }$, the values of NPCR and UACI are calculated according to Equation (24). After 200 pairs values of NPCR and UACI are obtained in the same way, the average of NPCR and UACI will be obtained, which is shown in Table 6. As one can observe from Table 6, all calculation results are close to the theoretical values. Furthermore, eight standard images with different sizes are used to perform randomness tests and all standard images pass the randomness test as Table 7 and Table 8 show [42].

### 4.5. Known and Chosen Plaintext Analysis

**Figure 7:**
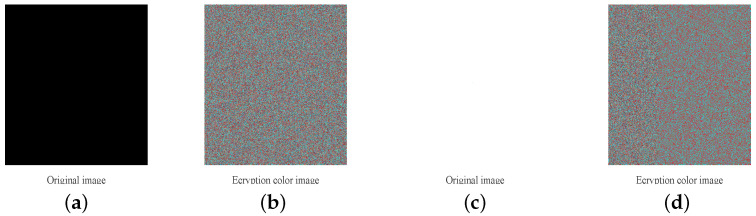
Encryption result of special images: (**a**) black image; (**b**) the encrypted black image; (**c**) white image; (**d**) the encrypted white image.

Known/chosen plaintext analysis are powerful cryptanalysis approaches used by attackers. Some special plain images such as all black or all white images are chosen or constructed by attackers and used to obtain the corresponding encrypted images to deduce the key streams (even the secret key) or disclose the relation between plain image and encrypted image. In our scheme, however, the generation of the values of the parameters of cat map that not only used the permutation stage but used it to calculate the value of the first encrypted pixels $C_{R}\left(1\right)$, $C_{G}\left(1\right)$, $C_{B}\left(1\right)$ is related to the plain image. Thus, it means that both the permutation stage and diffusion stage are related to plain image, which can obtain high key sensitivity and plaintext sensitivity to resist chosen/known plaintext attacks effectively. The encryption results of all black and all white images are shown in Figure 7 and one can observe that all cipher images are noise-like. Furthermore, we construct two other special plain images denoted as $P_{1}$ and $P_{2}$. $P_{1}$ is a color image with size M×N×3 in which all pixels’ values are zero except one pixel located in (250,250) in R channel is 1. $P_{2}$ is also a color image with size M×N×3 in which all pixels’ values are 255 except for one pixel located in (250,250) in the R channel, which is 0. Then, we use the four special plain images to do plaintext sensitivity analysis and the analysis results are shown in Table 9. As shown in Table 9, the average values of NPCR and UACI are almost equal to the theoretical value.

### 4.6. Robustness against Noise and Occlusion Attacks

**Figure 8:**
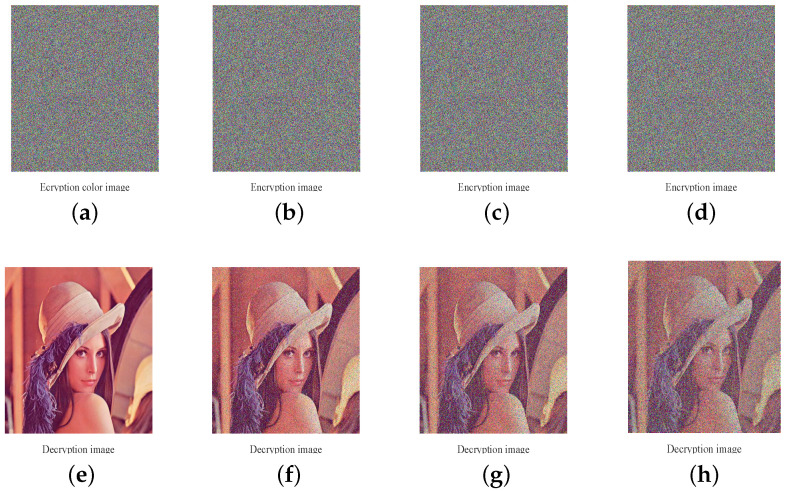
Robustness against noise results: (**a**,**e**): encrypted image and its decrypted image; (**b**,**f**): encrypted image added with salt and pepper noise with 0.1 density and corresponding decrypted image; (**c**,**g**): encrypted image added with salt and pepper noise with 0.2 density and its decrypted image; (**d**,**h**): encrypted image added with salt and pepper noise with 0.3 density and its decrypted image.

**Figure 9:**
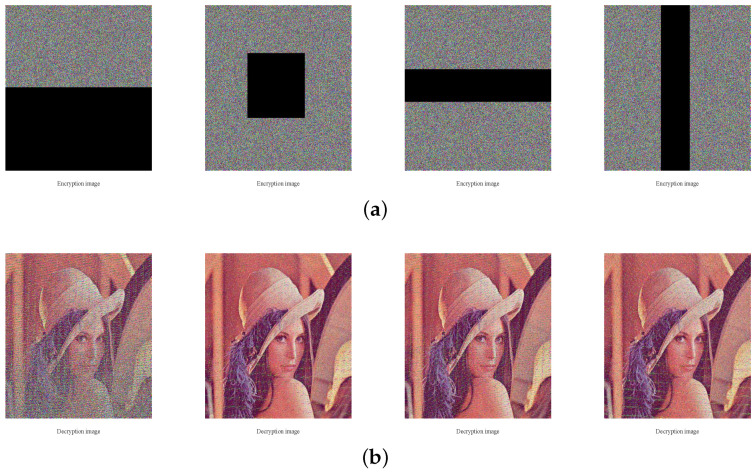

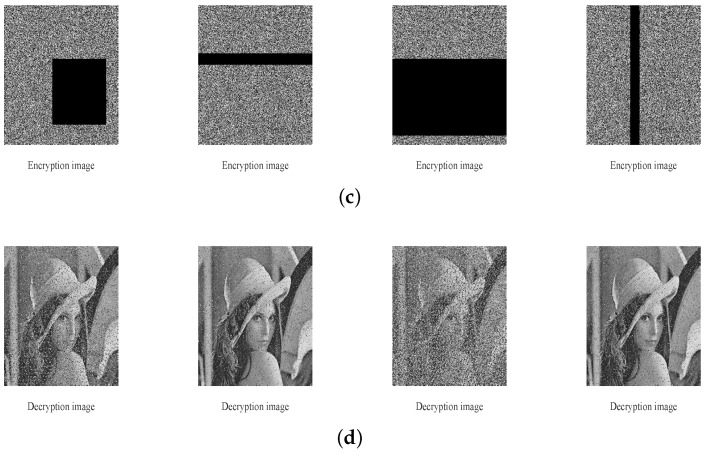
Robustness analysis results. (**a**) The color cipher images of lena with different percentages data loss; (**b**) The corresponding decrypted images of (**a**); (**c**) The gray cipher images of lena with different percentages data loss; (**d**) The corresponding decrypted images of (**c**).

When the encrypted images are transmitting through the public network, it is easily contaminated by noise or occlusion-attack. In this section, we use standard color image Lena with size 512×512 to test the robustness to resist noise and the occlusion attack. As Figure 8 shows, the decrypted images of encrypted images polluted by different densities salt-and-pepper noise can still recognized. In Figure 9, the decrypted images of color or gray cipher images destructed with different degrees can also still be recognized. Thus, our image cryptosystems have strong robustness to resist against noise attack and occlusion attack. Furthermore, PSNR (Peak Signal to Noise Ratio) is often used to evaluate the restoring ability of an image and expressed using the following equation [43,44]:

(26) $$PSNR=10\times \log\frac{255^{2}}{MSE}\left(dB\right),$$

where $MSE=\frac{1}{3\times M\times N}\sum_{i=1}^{N}\sum_{j=1}^{M}\sum_{k=1}^{3}{\left(O(i,j,k)-D(i,j,k)\right)}^{2}$, M and N are the size of the image, *O* is the original image and *D* is the decrypted image of the cipher image contaminated by noise or occlusion-attack. The larger the value of PSNR, the less distortion of the image. Here, we use standard gray image Lena with size 256×256 as a testing image and Table 10 shows the PSNR analysis results of our scheme compared with a plain related image encryption scheme in Ref. [35]. As Table 10 shows, the performance of robustness to resist noise and the occlusion attack of the proposed encryption algorithm is better than the one in Ref. [35].

### 4.7. Information Entropy

In this section, we use an important index of information entropy to measure gray values of an image’s unpredictability and randomness. For a 256 gray-scale image, information entropy is calculated quantitatively with

(27) $$H\left(m\right)=\sum_{i=0}^{255}p(m_{i})\log\frac{1}{p(m_{i})},$$

where *m* is a 256 gray-scale image. For the digital image with 256 gray levels, the information entropy is equal to a theoretical value of 8 when different gray level pixels appear randomly. Table 11 shows the values obtained for the entropies of standard original images and its cipher-images. One can observe from Table 11 that all information entropies of cipher-images, as expected, are close to 8. Therefore, the distribution of different gray level pixels is very uniform, which means that the proposed image cryptosystem has better ability to resist statistical attacks.

### 4.8. Encryption Speed Analysis

In this section, speed analysis is given and some similar plaintext related algorithms in Ref. [28,32,36,38] are used to make a comparison with our scheme. Here, one standard image of Lena with size 256×256 or 512×512 is used as a test image and the running speed of different algorithms in literature are listed in in Table 12. It should be noted that our experimental environment is MATLAB R2014b (MathWorks, Natick, MA, USA) with Intel Core i7-7500U CPU@ 3.5 GHz (Intel, Santa Clara, CA, USA) and 4.0 G RAM on Windows 10 OS (Microsoft, Redmond, WA, USA) and the results of encryption time consumption include both key-stream generation and encryption operations.

Furthermore, encryption throughput (ET) in Mega Byte Per Second (MBps) and number of cycles per byte defined by Equations (28) and (29) are also given to evaluate the encryption speed of our cryptosystem:

(28) $$ET=\frac{{\mathrm{Image}}_{Size}\left(Byte\right)}{Encryption_{Time}\left(second\right)},$$

(29) $$Number\;of\;cycles\;per\;Byte=\frac{CPU\;Speed_{\left(Hertz\right)}}{ET_{\left(Byte\right)}}.$$

As Table 12 and Table 13 show, compared to most of other algorithms, our scheme has more efficiency.

## 5. Conclusions

To conquer the issue of low key sensitivity and plaintext sensitivity in most encryption schemes proposed recently and obtain a high security and efficient encryption system, a simple chaotic based color image encryption system using both plaintext related permutation and diffusion is presented. In the permutation stage, the values of the parameters of cat map are related to plain images. It means that different original images correspond to different parameters. Thus, the permutation stage is related to plain image. In the diffusion stage, the first encrypted pixels’ value is determined by both secret keys and the parameter of cat map. Furthermore, we use previously encrypted pixels to encrypt current encrypting pixels. Thus, the diffusion stage is also related to plain image, which can achieve high key sensitivity and plaintext sensitivity to resist chosen/known plaintext attacks or differential attacks effectively. In addition, plaintext related permutation and diffusion operation is performed for only one round to process the original image to obtain the cipher image. Thus, our scheme has high efficiency. Complete simulations are given and the experimental results prove that the proposed scheme has high robustness to resist brute-force attacks, statistical analysis, differential attacks, noise attacks, occlusion attacks and the powerful chosen/known plaintext attacks. Thus, our scheme can be used to encrypt digital image efficiently.

## Figures and Tables

**Table 1:** Some cryptosystems attacked by some typical approaches.

Cryptosystems	Attacked by	Attack Approaches
Zhang et al. (2013) [4]	Hoang et al. (2018) [21]	chosen ciphertext
Zhou et al. (2015) [6]	Chen et al. (2017) [22]	Differential
Zhang et al. (2016) [7]	Wu et al. (2018) [23]	chosen plaintext
Huang et al. (2012) [9]	Wang et al. (2014) [24]	chosen plaintext
Chen et al. (2015) [10]	Hu et al. (2017) [25]	chosen plaintext and ciphertext
Liu et al. (2016) [14]	Zhang et al. (2017) [26]	chosen plaintext
Pak et al. (2017) [18]	Wang et al. (2018) [27]	chosen plaintext

**Table 2:** Variance of the histogram of cipher image (R channel).

Image	Lena	Baboon	Flower	Fruits	Yacht	Girl	Flowers
Variance	898.25	1017.29	1095.71	899.87	917.07	1413.86	783.00

**Table 3:** Average correlation coefficients of the original images and the cipher-images in four directions.

Image	Original-Image					Encrypted-Image			
		V	H	D	A	V	H	D	A
Lena	R	0.9798	0.9893	0.9777	0.9697	0.0003	0.0040	0.0013	0.0021
	G	0.9689	0.9824	0.9653	0.9554	−0.0018	0.0005	0.0002	0.0009
	B	0.9325	0.9574	0.9253	0.9181	0.0019	−0.0093	0.0002	0.0005
baboon	R	0.9231	0.8660	0.8519	0.8543	0.0002	0.0019	−0.0005	0.0022
	G	0.8654	0.7650	0.7249	0.7348	−0.0019	−0.0046	0.0040	0.0010
	B	0.9072	0.8808	0.8424	0.8398	−0.0039	−0.0062	0.0013	0.0020
fruits	R	0.9936	0.9928	0.9897	0.9868	−0.0022	−0.0021	−0.0027	0.0010
	G	0.9855	0.9848	0.9783	0.9694	0.0069	0.0073	0.0021	−0.0016
	B	0.9265	0.9192	0.8809	0.8531	0.0007	0.0088	−0.0003	−0.0010
flowers	R	0.9718	0.9719	0.9504	0.9551	0.0045	−0.0002	−0.0005	0.0007
	G	0.9510	0.9497	0.9123	0.9218	0.0047	−0.0015	0.0026	0.0022
	B	0.9527	0.9527	0.9178	0.9256	0.0004	−0.0032	0.0019	−0.0008

**Table 4:** Correlation coefficients comparison for different encryption algorithms (R channel of Lena).

Direction	Original Image	Our Scheme	Ref. [11]	Ref. [18]	Ref. [28]	Ref. [32]	Ref. [36]
Horizontal	0.9853	0.0003	0.0013	−0.0038	−0.0031	0.0046	0.0005
Vertical	0.9753	0.0040	0.0034	−0.0026	0.0025	−0.0028	−0.0070
Diagonal	0.9734	0.0013	0.0072	0.0017	−0.0001	0.0014	0.0006

**Table 5:** Evaluation results of the key sensitivity using NPCR and UACI.

Image	NPCR (99.6094)				UACI (33.4635)		
		R	G	B	R	G	B
Lena	$key_{\_x0}$	99.6089	99.6089	99.6085	33.4589	33.4598	33.4624
	$K_{1}$	99.6092	99.6075	99.6095	33.4615	33.4676	33.4624
	$K_{2}$	99.6082	99.6101	99.6087	33.4626	33.4670	33.4686
	$K_{3}$	99.6087	99.6094	99.6098	33.4609	33.4623	33.4628
	$N_{0}$	99.6090	99.6091	99.6090	33.4662	33.4619	33.4684
baboon	$key_{\_x0}$	99.6086	99.6096	99.6106	33.4639	33.4621	33.4641
	$K_{1}$	99.6107	99.6089	99.6093	33.4641	33.4651	33.4696
	$K_{2}$	99.6083	99.6103	99.6093	33.4685	33.4653	33.4620
	$K_{3}$	99.6087	99.6112	99.6087	33.4616	33.4636	33.4593
	$N_{0}$	99.6096	99.6090	99.6087	33.4686	33.4645	33.4662

**Table 6:** Evaluation results of the plain-image sensitivity using NPCR and UACI.

Image	NPCR (99.6094)			UACI (33.4635)		
	R	G	B	R	G	B
Lena	99.6091	99.6099	99.6090	33.4678	33.4577	33.4608
baboon	99.6111	99.6097	99.6094	33.4617	33.4680	33.4617
fruits	99.6091	99.6081	99.6091	33.4631	33.4663	33.4593
Girl	99.6090	99.6100	99.6095	33.4627	33.4597	33.4588
Flower	99.6113	99.6098	99.6100	33.4603	33.4666	33.4588
Yacht	99.6092	99.6095	99.6099	33.4613	33.4666	33.4651
Lena in Ref. [11]	99.6892	99.6943	99.6922	33.3256	33.3324	33.3313
Lena in Ref. [18]	99.6552	99.6277	99.5882	33.4846	33.4132	33.3441
Lena in Ref. [28]	99.6917	99.6887	99.6704	33.5418	33.5327	33.5164
Lena in Ref. [32]	99.6086	99.6083	99.6104	33.4709	33.4683	33.4682

**Table 7:** NPCR randomness test.

	**Theoretically NPCR Critical Value [42]**		
	$N_{0.05}^{*}=99.5893$	$N_{0.01}^{*}=99.5810$	$N_{0.001}^{*}=99.5717$
**Tested Image Size**	**NPCR Test Results**		
	**0.05-level**	**0.01-level**	**0.001-level**
512 by 512	4/4	4/4	4/4
256 by 256	4/4	4/4	4/4

**Table 8:** UACI randomness test.

	**Theoretically UACI Critical Value [42]**		
	$u_{0.05}^{*-}=33.3730$ $u_{0.05}^{*+}=33.5541$	$u_{0.01}^{*-}=33.3445$ $u_{0.01}^{*+}=33.5826$	$u_{0.001}^{*-}=33.3115$ $u_{0.001}^{*+}=33.6156$
**Tested Image Size**	**UACI Test Results**		
	**0.05-level**	**c0.01-level**	**0.001-level**
512 by 512	4/4	4/4	4/4
256 by 256	4/4	4/4	4/4

**Table 9:** NPCR and UACI indicators for special plaintexts.

Original Image	NPCR (99.6094)			UACI (33.4635)		
	R	G	B	R	G	B
All-black	99.6121	99.6102	99.6105	33.5744	33.5200	33.3917
All-white	99.6075	99.6100	99.6100	33.4578	33.4683	33.4555
$P_{1}$	99.5284	99.4976	99.4469	33.4466	33.6076	33.3642
$P_{2}$	99.6104	99.6089	99.6113	33.4254	33.5033	33.4453

**Table 10:** Results of noise and occlusion attacks.

Noise Attacks or Date Loss	MSE (Proposed)	PSNR (Proposed)	MSE (Ref. [35])	PSNR (Ref. [35])
Salt & peppers noise (density 0.05)	734.3922	19.4715	869.8890	18.7362
Salt & pepper noise (density 0.1)	1465.4644	16.4711	1829.6416	15.5071
(100:220,110:230)=0	1729.6835	15.7511	2894.6596	13.5148
(90:110,:)=0	658.2202	19.9471	1073.0810	17.8245
(100:240,:)=0	4335.8104	11.7601	6813.5770	9.7971
(:,100:120)=0	632.5614	20.1198	946.0235	18.3718

**Table 11:** Information entropy of some standard images encrypted by different algorithms.

Image	Plain-Image			Encrypted Image		
	R	G	B	R	G	B
Lena	7.2531	7.5952	6.9686	7.9993	7.9994	7.9994
baboon	7.7067	7.4753	7.7522	7.9993	7.9993	7.9993
fruits	7.5172	7.3230	6.7785	7.9991	7.9993	7.9991
flower	7.4428	7.4062	7.3371	7.9993	7.9994	7.9994
Girl	7.4346	7.2354	7.0578	7.9996	7.9995	7.9995
Yacht	7.6071	7.4062	7.3371	7.9993	7.9993	7.9991
Lena in Ref. [11]	7.2531	7.5952	6.9686	7.9996	7.9997	7.9997
Lena in Ref. [28]	7.2531	7.5952	6.9686	7.9972	7.9972	7.9976
Lena in Ref. [32]	7.2531	7.5952	6.9686	7.9992	7.9993	7.9994

**Table 12:** Encryption time (seconds) for color image.

Image Size	Proposed Method	Ref. [28] (2015)	Ref. [32] (2018)	Ref. [36] (2017)	Ref. [38] (2017)
512×512	4.4113	3.0080	4.6058	14.8119	21.1786
256×256	1.0896	0.6650	1.1347	3.6175	4.7795

**Table 13:** Encryption throughput and number of cycles for one encrypted byte.

Scheme	ET in MBps	Number of Cycles Per Byte
Proposed	0.170	19634.47
Mollaeefar et al. (2015) Ref. [28]	0.249	13405.06
Cai et al. (2018) Ref. [32]	0.165	20229.45
Wu et al. (2018) Ref. [36]	0.050	66757.20
Luo et al. (2017) Ref. [38]	0.035	95367.43
